# The Surgical Site Infection Risk Score (SSIRS): A Model to Predict the Risk of Surgical Site Infections

**DOI:** 10.1371/journal.pone.0067167

**Published:** 2013-06-27

**Authors:** Carl van Walraven, Reilly Musselman

**Affiliations:** 1 Department of Medicine, University of Ottawa, Ottawa, Canada; 2 Department of Surgery, University of Ottawa, Ottawa, Canada; 3 Ottawa Hospital Research Institute, Ottawa, Canada; 4 Institute for Clinical Evaluative Sciences, Toronto, Canada; Royal Melbourne Hospital, Australia

## Abstract

**Background:**

Surgical site infections (SSI) are an important cause of peri-surgical morbidity with risks that vary extensively between patients and surgeries. Quantifying SSI risk would help identify candidates most likely to benefit from interventions to decrease the risk of SSI.

**Methods:**

We randomly divided all surgeries recorded in the National Surgical Quality Improvement Program from 2010 into a derivation and validation population. We used multivariate logistic regression to determine the independent association of patient and surgical covariates with the risk of any SSI (including superficial, deep, and organ space SSI) within 30 days of surgery. To capture factors particular to specific surgeries, we developed a surgical risk score specific to all surgeries having a common first 3 numbers of their CPT code.

**Results:**

Derivation (n = 181 894) and validation (n = 181 146) patients were similar for all demographics, past medical history, and surgical factors. Overall SSI risk was 3.9%. The SSI Risk Score (SSIRS) found that risk increased with patient factors (smoking, increased body mass index), certain comorbidities (peripheral vascular disease, metastatic cancer, chronic steroid use, recent sepsis), and operative characteristics (surgical urgency; increased ASA class; longer operation duration; infected wounds; general anaesthesia; performance of more than one procedure; and CPT score). In the validation population, the SSIRS had good discrimination (c-statistic 0.800, 95% CI 0.795–0.805) and calibration.

**Conclusion:**

SSIRS can be calculated using patient and surgery information to estimate individual risk of SSI for a broad range of surgery types.

## Introduction

Surgical site infections [SSIs] are important events. They are one of the most common nosocomial infections [1], occurring in 2–5% of the estimated 30 million operations occurring annually in the United States [2]. They are associated with significantly increased health care costs [3]. Most importantly, they have important implications for patients by causing pain, increasing the risk of hospital readmissions, and making repeated procedures more likely [4].

Being able to accurately quantify SSI risk would be helpful for two primary reasons. First, determining the likelihood that a particular patient develops an SSI is essential for deciding whether or not particular preventive strategies [such as prophylactic antibiotics] should be used. This is because the probability that a particular patient benefits from such strategies is inversely associated with baseline risk of the event. Second, an accurate risk model would facilitate the comparison of SSI rates between facilities and health care providers.

A large number of SSI risk models that are specific to particular surgery types have been published. One of the most common tools to predict SSI risk in a broad range of surgeries is the National Nosocomial Infections Surveillan [NNIS] Basic SSI Risk Index [5]. This model has several limitations including: its small number of potential final scores [thereby limiting its discriminatory abilities]; the uncertain validity of equally weighting all three components of the model; and its inability to risk stratify particular specific surgeries [6], [7]. Mu et. al. [8] created 39 procedure-specific models with more extensive candidate variables and greater c-statistics than the NNIS Basic SSI Risk Index [median values of 0.67 vs. 0.60] but were still weakly discriminative.

In this study, we derived and internally validated a model to predict the risk of developing surgical site infections [SSIs] within 30 days of surgery. SSI risk for an individual patient can be estimated with this model via a webpage that we have developed or using a point system created from the model.

## Methods

### Data Source

This study used data from the American College of Surgery National Surgical Quality Improvement Program [ACS-NSQIP]. ACS-NSQIP collects prepoperative data and 30-day morbidity outcomes on patients undergoing major operations meeting program criteria. Data are collected by uniformly trained Surgical Clinical Reviewers [SCR] and entered into a secure internet website. Data are subjected to quality checks with detailed examination for inter-rater reliability.

Cases were collected in 8-day cycles with participating hospitals either: including all specialties; or limiting their cases to general/vascular surgery. Cases were sampled by the SCR from each institution’s operative log and were excluded if: the patient was less than 18 years old; surgical indications included acute trauma, transplantation, or brain-dead organ donor; the procedure was an inguinal herniorrhaphy, breast lumpectomy, laparoscopic cholecystectomy, or transurethral resection of the prostate and three examples of the procedure had already been sampled within the current 8-day sampling cycle; or the patient had already been included within the previous 30 days. In high-volume hospitals, the first consecutive general and vascular surgeries that qualified within each cycle were included while approximately 20% of cases from other specialties were included [varying by hospital size]. Low-volume hospitals were required to enter at least 22 general/vascular and other specialty cases per cycle.

Standard data collection sheets were used at each centre. The patient’s electronic and/or paper medical record was reviewed to abstract all preoperative and intraoperative information. Post-operative information was collected by review of the in-patient medical record, out-patient charts, and phone calls or letters to the patient.

### Analytical Dataset: Population, Primary Outcome, and Covariates

The data were collected by the National Surgical Quality Improvement Program. These data are collected without explicit, written patient consent since they are for quality improvement. Our study was reviewed by the NSQIP team [the data owners], who released both patient- and institutional- deidentified data to us for our analyses. We used the Participant Use Data File from ACS-NSQIP which contained all reported surgeries from participating hospitals in 2010 and excluded all operations containing maligned CPT codes. The study was approved by the Ottawa Hospital Research Ethics Board.

The primary outcome was surgical site infection [SSI] within 30 days of operation. This outcome included all patients with a superficial, a deep, or an organ-space infection [Appendix S1]. In the presence of an open wound, SSIs were counted only if they were initially detected more than two days after the operation. We considered all pre- and intra-operative covariates collected by NSQIP as long as data for the covariate was missing in less than 1% of cases [thereby excluding pre-operative laboratory information, since these were missing in at least ∼15% of patients].

### Analysis

The cohort was randomly divided into equally sized derivation and validation groups. Non-linear associations between continuous variables and the outcome were identified using fractional polynomials [9], [10].

In the first stage of modeling, we used binomial logistic regression [SAS 9.2, Cary NC] with forward variable selection to identify the covariates independently associated with 30-day SSI status. This model was then used to gauge SSI risk *within* surgical groups independent of all covariates in the initial model. Surgeries that had the same first three numbers in their CPT code were grouped together. SSI risk within each surgical group was quantified as the ratio of the observed to expected number of SSIs [with the latter calculated from the initial model]. We called this statistic the “CPT3 Score” in which scores below 1 indicated that fewer than expected SSIs were observed based on the patient population. If no SSIs were observed within a surgical group, we assigned CPT3 Scores of 0 if the *expected* number of SSIs in the group exceeded 0.5 [otherwise, CPT3 Scores were defaulted to 1].

In the second stage of modeling, we added the CPT3 Score to all other covariates and used binomial logistic regression with forward selection to identify all covariates independently and strongly [p<0.0001] associated with SSI. Finally, we tested the significance of several potential interactions identified *a priori* based on clinical criteria.

We used methods described by Sullivan *et al* [11] to convert the model to the “SSI Risk Score” [SSIRS]. We calculated the expected risk change associated with each SSIRS point using a logistic regression model having 30-day SSI as the outcome and risk score as the only dependent variable.

The model and SSIRS was evaluated by determining both discrimination [using the c-statistic with 95% confidence intervals [12]] and calibration [using the Hosmer-Lemeshow goodness-of-fit test [13]]. The c-statistic is equivalent to the area under the receiver operating characteristic [ROC] curve. Numerically, it is the proportion of time that the estimated risk from a model is higher in the person with an event vs. the person without an event. A valued of 0.5 indicates that the model is nor better than chance at predicting risk. Models are typically considered reasonable when the C-statistic exceeds 0.7 and strong when it exceeds 0.8 [14].

We further assessed calibration by comparing the expected to observed event rate at each SSIRS vallue. The expected risk of SSI for each patient was calculated as the inverse of 1+ *e* ^−[intercept+β*SSIRS value]^, where β was the coefficient of the risk score in a logistic regression model whose sole covariate was SSIRS. The expected and observed event rates were considered similar if the expected rate was within the exact 95% CI around the observed rate [15]. We compared the performance of our model with that of the NNIS basic risk model [5].

## Results

There were 363 431 surgeries recorded in NSQIP during 2010. 391 [0.1%] of these were excluded because of invalid CPT codes leaving 363 040 surgeries in the study [181 894 for model derivation, 181 146 for model validation].

The study cohort is summarized in Table S1 [a complete description of the cohort - including all candidate variables for the model - is provided in Appendix S2]. Patients were middle aged and predominantly independent. Approximately one third of surgeries were ambulatory, one half were elective in-patient surgeries, and the remaining 10% were emergency surgeries. More than half of surgeries had clean fields and more than half of patients had ASA scores of 1 or 2. Surgeries involved general anaesthesia more than 90% of the time, housestaff more than half of the time, and an additional procedure [by the same team] more than a third of the time. Mean duration of the operation was 1.8 hours. Derivation and validation groups were essentially identical [Table S1].

Overall, SSIs occurred after 14 227 surgeries [3.9%]. The majority of these [n = 8188, 57.5% of all SSIs] had a superficial component while 15.8% and 29.2% of SSIs involved the deep incision or the organ space, respectively. Overall SSI risk, as well as SSI type, was similar in the derivation and validation groups. SSI risk increased significantly with increased levels in the NNIS basic risk model [Table S2]. 82% of the cohort had an NNIS score of 0 or 1, resulting in weak discrimination [c-statistic 0.641].Covariates describing the actual surgery predominated in the initial model [Appendix S3]. This initial model included 5 of the 11 variables dealing with patient demographics [45.4%], 10 of 32 variables describing patient medical history [31.2%], and 9 of 11 variables [81.8%] describing surgical information. The initial model had adequate discrimination [C-statistic 0.772].

The initial model was used to generate the CPT3 Score [Appendix S4]. Of the 296 possible unique combinations of the first 3 numbers of CPT codes: 288 [97.3%] had at least one operation in the derivation group; 48 [16.2%] had no observed SSIs but had an expected number of SSIs [based on the initial model] that exceeded 0.5 [and were therefore were assigned a CPT3 Score of “0”]; and 56 [18.9%] had no observed SSIs but had an expected number of SSIs [based on the initial model] of less than 0.5 [and were therefore assigned a CPT3 Score of “1”]. Overall, CPT3 Scores ranged from 0 to 4.07 with a mean value of 0.895 and median value of 0.984 [Interquartile Range 0.394–1.086].

In the final model, the CPT3 Score was strongly and independently associated with 30-day SSI risk along with a dozen other covariates and four interactions [Table S3]. SSI risk increased with: smoking; increased body mass index; peripheral vascular disease; metastatic cancer; steroid use; and pre-operative sepsis. Procedural factors that notably increased the risk of infection included: inpatient and emergent setting; contaminated or dirty operative fields; ASA scores of 3 or more; the use of a general [instead of local] anesthesia; the conduct of more than one procedure during the surgery; and increased operative time. In the validation group, the model had both excellent discrimination [C-statistic = 0.800 [95% CI 0.795–0.805]]. Our web-page can be used to calculate SSI risk for a particular patient from this model [http://www.ohri.ca/SSI_risk_index/Default.aspx ].

The model was modified into the SSI Risk Score [SSIRS] to make risk calculation without a computer possible [Table S4]. Each SSIRS point equaled the increased SSI risk associated with a 5-unit increase of the body mass index. Categorical variables independently associated with a notably increased risk of an SSI included: a contaminated/dirty or infected wound; inpatient and emergency surgery; and an ASA score exceeding 3. Notably influential continuous covariates included: a BMI exceeding 35; operation duration less than or equal to ½ hour or exceeding 3½ hours; and a CPT3 score less than 0.9 or exceeding 1.262.

SSIRS had a potential range between −17 and 62 but had an observed range between −15 and 50 [median score: 13, interquartile range: 7 to 21] [Figure S1]. Discrimination using SSIRS was very good [c-statisitc 0.781, 95% CI 0.776–0.786] but was significantly lower than that of the entire model [Table S3]. The predicted SSI risk based on SSIRS was within the 95% confidence interval of the observed risk in 49 of the 58 point levels in which an SSI was observed [84.5%] capturing 89.7% of the validation population.

## Discussion

This study derived and internally validated a model that uses commonly available information to predict the risk of SSI within 30-days of an operation. Compared to the NNIS Basic Risk model, it had significantly better discrimination while maintaining adequate calibration. The model was also modified to the SSI Risk Score [SSIRS] that permits 30-day SSI risk to be estimated at the bedside without computational aids. If validated in an external patient population, this model will be a significant advance for predicting the risk of SSI in patients having surgeries.

Determining SSI risk has two important applications. First, accurate quantification of SSI risk is needed to compare SSI rates between patients groups [defined by hospitals, clinical services, or individual surgeons]. Second, determining SSI risk for an individual patient is necessary to gauge the potential utility of preventive interventions. For example, an intervention that halves SSI risk [slightly less than the risk reduction associated with prophylactic antibiotics [16]] has a number needed to treat [NNT] to avoid one SSI of 20 when the baseline risk is 10% but a NNT of 200 when the baseline risk is 1%. Surgeon behaviour and practice guidelines reflect the fact that higher-risk patients are more likely to benefit from preventive interventions since antibiotic prophylaxis is recommended for patients with contaminated or dirty wounds – patients who have a higher risk of SSI. Our model illustrates that many other factors contribute to SSI risk. Systematically considering all of these factors should result in a more accurate estimate of SSI risk and better decisions regarding SSI preventive therapies.

Our model was similar to, but distinct from, other studies examining SSI risk in patients. The CPT3 Score in our model used an approach similar to that by Raval *et al* [17], who used a more clinically robust method to cluster CPT codes into procedural groups. Multiple previous studies have found significant associations between the risk of SSI and factors in our model [including wound class, body mass index, surgical location and urgency, ASA class, the performance of more than one procedure, metastatic cancer, the presence of steroids, and surgical duration] [18]. One study found that smoking [19] is an independent predictor of SSI in breast cancer surgeries. We believe that our study is innovative in the way it gauged the independent influence that each of these factors – along with a new method of quantifying the influence of different operative types – have on SSI risk.

Our model has several notable strengths. It was derived and internally validated on a large cohort of patients involving a large number and a broad range of hospitals, surgeons, and surgeries. The analytical dataset included prospectively collected data with explicitly defined covariates. Our model was very accurate with both excellent discrimination and calibration. Finally, the model has real practical relevance to clinicians and patients since it permits the SSI risk for a particular patient to be calculated through the internet [http://www.ohri.ca/SSI_risk_index/Default.aspx] or through the SSI Risk Index [Table S4].

Several limitations of our study should be noted when interpreting its results. The most important limitation to our model is its inability to capture interventions for reducing SSI risk given to patients. The most important of these are peri-operative antibiotics, which has a relative risk of SSI that varies between 0.19 and 0.85 [16]. Given that they so effectively decrease SSI risk, and are more likely to be given to patients with more aggressive wounds, we believe that our model and index *underestimates* SSI risk associated with wounds other than clean wonds. The second most important limitation is that our model has not been externally validated. Until this has happened, people should use it cautiously to predict SSI risk in patients. Other limitations are notable: the model, when expressed as a scoring system, is too complicated to memorize [a notable benefit of the NNIS Basic Risk model]; the model requires that the user knows the CPT code of the surgery; one of the components of the model – duration of the operation – is unknown *prior* to the operation when interventions to decrease the risk of SSI would primarily be invoked. Therefore, SSI risk estimates *prior* to the operation would only be approximations since they would require an estimate of the operation duration.

In summary, we derived and internally validated a model that predicts the risk of 30-day SSI in a broad range of surgical procedures. These data show that the risk of SSI can be estimated using readily available covariates regarding the patient and the surgery. We believe that external validation of this model, especially with data that incorporate information regarding prophylactic antibiotics, should be pursued before this model is used to predict the risk of surgical site infections in individual patients.

## Supporting Information

Figure S1 **Relationship between the Surgical Site Infection (SSI) Risk Score (SSIRS) and 30-day SSI probability in the validation population.** For each SSI Risk Score value (horizontal axis), this graph presents: the observed number of people (columns, left vertical axis); the observed percentage of people with an SSI within 30-days of surgery (red line, right vertical axis); and the expected percentage of people with an SSI (black dotted line, right vertical axis). 95% confidence intervals are presented for the observed percentages.
(TIFF)

Table S1 **Description of study cohort.**
(DOC)

Table S2 **Performance of the NNIS Basic Risk Model in the study cohort.**
(DOC)

Table S3 **Model to predict 30-day risk of surgical site infection.**
(DOC)

Table S4 **SSI Risk Score (SSIRS).**
(DOC)

Appendix S1 **Definitions used for superficial, deep, and organ surgical site infections.**
(DOC)

Appendix S2 **Complete description of all covariates offered to model.**
(DOC)

Appendix S3 **Initial logistic model to predict risk of 30-day surgical site infection.**
(DOC)

Appendix S4 **The CPT3 Score.**
(DOC)
