# Supplementary figures and images for: The Surgical Site Infection Risk Score (SSIRS): A Model to Predict the Risk of Surgical Site Infections
Source: PLoS One. 2013 Jun 27;8(6):e67167. doi: 10.1371/journal.pone.0067167 (PMC3694979; doi:10.1371/journal.pone.0067167)

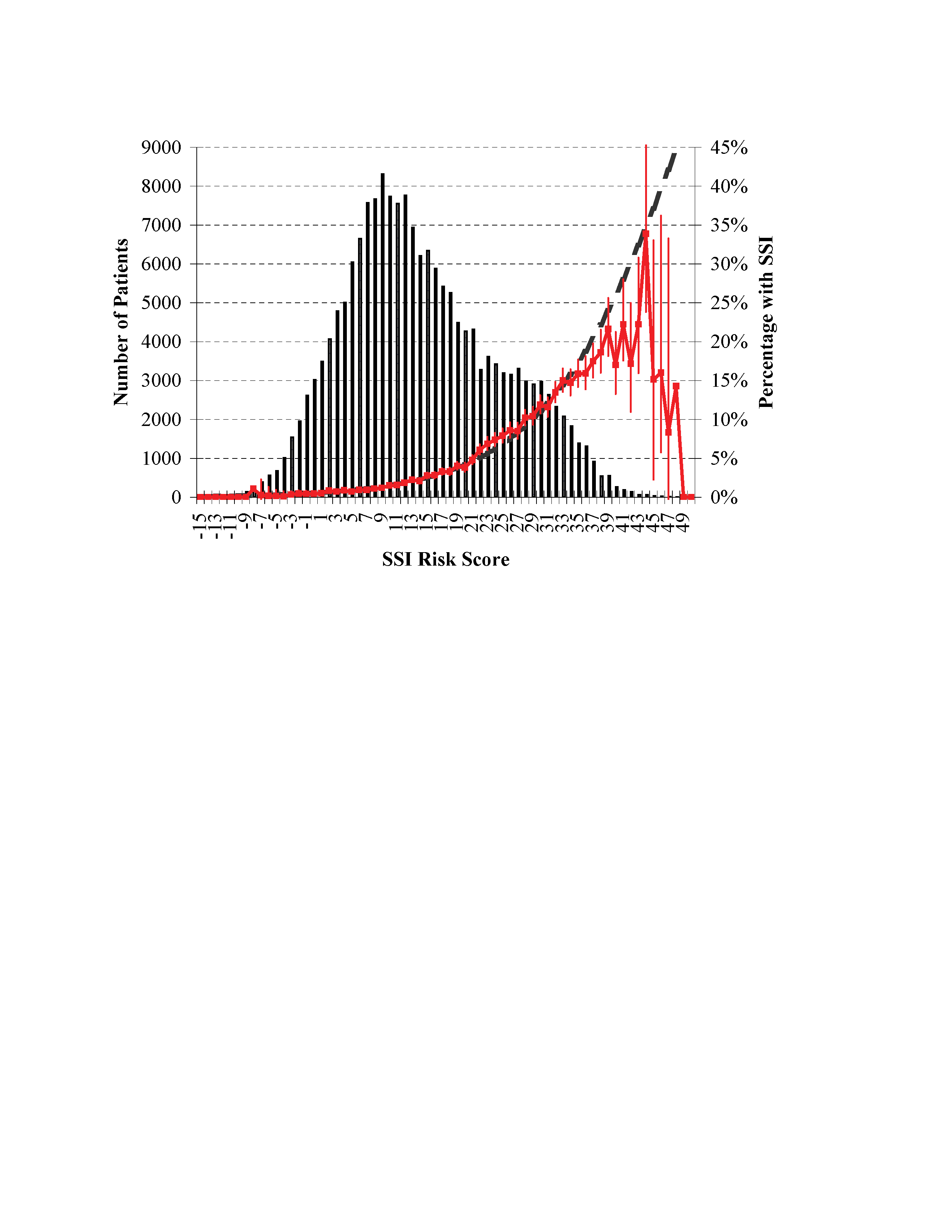

Supplement: Figure S1 — Relationship between the Surgical Site Infection (SSI) Risk Score (SSIRS) and 30-day SSI probability in the validation population. For each SSI Risk Score value (horizontal axis), this graph presents: the observed number of people (columns, left vertical axis); the observed percentage of people with an SSI within 30-days of surgery (red line, right vertical axis); and the expected percentage of people with an SSI (black dotted line, right vertical axis). 95% confidence intervals are presented for the observed percentages. (TIFF) [file pone.0067167.s001.tiff]
