# Supplementary material for: The Surgical Site Infection Risk Score (SSIRS): A Model to Predict the Risk of Surgical Site Infections
Source: PLoS One. 2013 Jun 27;8(6):e67167. doi: 10.1371/journal.pone.0067167 (PMC3694979; doi:10.1371/journal.pone.0067167)
Supplement: Table S1 — Description of study cohort. (DOC) [file pone.0067167.s002.doc]

**Table S1:** Description of study cohort.

|  | **DERIVATION** | **VALIDATION** | **OVERALL** |
| --- | --- | --- | --- |
|  | **N=181 894** | **n=181 146** | **n=363 040** |
| ***A - PATIENT DEMOGRAPHICS*** |  |  |  |
| Mean Age (SD) | 56 (16.9) | 55.9 (16.9) | 56 (16.9) |
| Male | 77 919 (42.8) | 77 138 (42.6) | 155 057 (42.7) |
| Smoker | 35 792 (19.7) | 35 526 (19.6) | 71 318 (19.6) |
| >10% body weight lost in last 6 months | 3334 (1.8) | 3338 (1.8) | 6672 (1.8) |
| Major surgical procedure in last month | 4915 (2.7) | 4790 (2.6) | 9705 (2.7) |
| **Functionality:**  Independent | 171 784 (94.4) | 171 137 (94.5) | 342 921 (94.5) |
| Assistance required from another | 7469 (4.1) | 7442 (4.1) | 14 911 (4.1) |
| Total assistance | 2641 (1.5) | 2567 (1.4) | 5208 (1.4) |
| Mean Body Mass Index in pounds per square inch (SD) | 30.1 (8.2) | 30 (8.1) | 30 (8.2) |
| ***B – PAST MEDICAL HISTORY*** |  |  |  |
| Previous stroke - with persistent deficit | 4004 (2.2) | 3937 (2.2) | 7941 (2.2) |
| - without deficit | 3683 (2.0) | 3590 (2.0) | 7273 (2.0) |
| Revascularization or amputation for PVD | 6850 (3.8) | 6885 (3.8) | 13 735 (3.8) |
| Rest pain or gangrene | 3895 (2.1) | 3867 (2.1) | 7762 (2.1) |
| Severe COPD* | 8912 (4.9) | 8907 (4.9) | 17 819 (4.9) |
| On ventilator within 48 hours of surgery | 1457 (0.8) | 1503 (0.8) | 2960 (0.8) |
| Metastatic cancer** | 3430 (1.9) | 3317 (1.8) | 6747 (1.9) |
| Steroid in last month for at least 10 days | 5072 (2.8) | 4949 (2.7) | 10 021 (2.8) |
| Bleeding diathesis, anticoagulant, or antiplatelet*** | 9570 (5.3) | 9415 (5.2) | 18 985 (5.2) |
| Radiation/brachytherapy in last 3 months | 1252 (0.7) | 1168 (0.6) | 2420 (0.7) |
| SIRS/sepsis in last 2 days | 13 679 (7.5) | 13 525 (7.5) | 27 204 (7.5) |
| ***C - SURGICAL INFORMATION*** |  |  |  |
| **Surgical Status:** Outpatient, non-emergency | 64 624 (35.5) | 64 695 (35.7) | 129 319 (35.6) |
| Inpatient, non-emergency | 98 101 (53.9) | 97 415 (53.8) | 195 516 (53.9) |
| Inpatient, emergency**** | 19 169 (10.5) | 19 036 (10.5) | 38 205 (10.5) |
| Open wound communicating directly with air | 7516 (4.1) | 7472 (4.1) | 14 988 (4.1) |
| **Wound Type**: Clean***** | 94 530 (52.0) | 94 354 (52.1) | 188 884 (52.0) |
| Clean/Contaminated | 63 052 (34.7) | 62 641 (34.6) | 125 693 (34.6) |
| Contaminated/Dirty, Infected | 24 312 (13.4) | 24 151 (13.3) | 48 463 (13.4) |
| **ASA Class:** 1 - normal healthy person | 17 182 (9.5) | 17 635 (9.7) | 34 817 (9.6) |
| 2 - mild systemic disease | 82 605 (45.4) | 82 585 (45.6) | 165 190 (45.5) |
| 3-5 - at least severe systemic disease | 82 107 (45.1) | 80 926 (44.7) | 163 033 (44.9) |
| General anaesthesia | 166 318 (91.4) | 165 726 (91.5) | 332 044 (91.5) |
| Resident not involved with operation | 84 895 (46.7) | 85 152 (47.0) | 170 047 (46.8) |
| Additional procedure by same surgical team | 67 059 (36.9) | 66 542 (36.7) | 133 601 (36.8) |
| Additional procedure by different surgical team | 6800 (3.7) | 6752 (3.7) | 13 552 (3.7) |
| Mean total operation time in hours (SD) | 1.8 (1.5) | 1.8 (1.5) | 1.8 (1.5) |

(SD = standard deviation) * Unable to perform activities of daily living; admitted previously for COPD; requiring chronic bronchodilators: FEV1<75% predicted. ** Solid tumor at >1 site; ALL; AML; or stage 4 lymphoma. *** With anticoagulant and antiplatelet not discontinued within specified time prior to operation. **** Required both surgeon and anaesthetist to state that surgery needed within 12 hrs. ***** No inflammation and respiratory/alimentary/genital/urinary tract not entered
