# Supplementary material for: The Surgical Site Infection Risk Score (SSIRS): A Model to Predict the Risk of Surgical Site Infections
Source: PLoS One. 2013 Jun 27;8(6):e67167. doi: 10.1371/journal.pone.0067167 (PMC3694979; doi:10.1371/journal.pone.0067167)
Supplement: Table S2 — Performance of the NNIS Basic Risk Model in the study cohort. (DOC) [file pone.0067167.s003.doc]

**Table S2:**  Performance of the NNIS Basic Risk Model in the study cohort

| **NNIS SCORE** | **N**  **(% of Cohort)** | **Observed SSIs (% of Strata)** | **Expected SSIs**  **(% of Strata)** |
| --- | --- | --- | --- |
| 0 | 134 438 (37.0%) | 2547 (1.9%) | 2650 (2.0%) |
| 1 | 163 410 (45.0%) | 6462 (4.0%) | 6317 (3.9%) |
| 2 | 58 808 (16.2%) | 4394 (7.5%) | 4377 (7.4%) |
| 3 | 6384 (1.8%) | 824 (12.9%) | 884 (13.9%) |
