# Supplementary material for: The Surgical Site Infection Risk Score (SSIRS): A Model to Predict the Risk of Surgical Site Infections
Source: PLoS One. 2013 Jun 27;8(6):e67167. doi: 10.1371/journal.pone.0067167 (PMC3694979; doi:10.1371/journal.pone.0067167)
Supplement: Table S3 — Model to predict 30-day risk of surgical site infection. (DOC) [file pone.0067167.s004.doc]

**Table S3:** Model to predict 30-day risk of surgical site infection.

| **Variable** | **Class Level** | **Estimate** | **Wald χ2** | **Adj. Odds Ratio** | **95% CI** |
| --- | --- | --- | --- | --- | --- |
| Intercept |  | -7.0222 | 2870.952 |  |  |
| ***A - PATIENT DEMOGRAPHICS AND BASELINE*** |  |  |  |  |  |
| Smoker |  | 0.1888 | 40.5 | 1.21 | (1.14, 1.28) |
| Body Mass Index |  | 0.017 | 159.7 | 1.02 | (1.01, 1.02) |
| ***B – PAST MEDICAL HISTORY*** |  |  |  |  |  |
| PVD requiring revascularization or amputation |  | 0.654 | 103.6 | - | - |
| Metastatic cancer*** |  | 0.236 | 14.2 | 1.26 | (1.20, 1.50) |
| Steroid in last month for at least 10 days |  | 0.260 | 20.4 | 1.30 | (1.16, 1.45) |
| SIRS/Sepsis in last 2 days |  | 0.514 | 29.7 | - | - |
| ***C - SURGICAL INFORMATION*** |  |  |  |  |  |
| Location, urgency (vs outpatient, non-emergency) | Inpatient, non-emergency | 0.244 | 7.5 | - | - |
|  | Inpatient, emergency | 0.908 | 50.5 | - | - |
| Wound Type (vs Clean) | Clean / Contaminated | 0.500 | 99.3 | - | - |
|  | Contaminated - Dirty | 1.025 | 344.1 | - | - |
| ASA Physical Status Classification (vs 1) | 2 (Mild Disturbance) | 0.358 | 25.4 | 1.43 | (1.24, 1.64) |
|  | 3+ | 0.601 | 72.0 | 1.82 | (1.59, 2.10) |
| General Anaesthesia |  | 0.260 | 12.0 | 1.30 | (1.12, 1.50) |
| Additional procedure by same surgical team |  | 0.210 | 61.7 | 1.23 | (1.17, 1.30) |
| Log (total operation in hours) |  | 0.613 | 310.5 | - | - |
| CPT3 Score |  | 0.938 | 273.6 | - | - |
| ***INTERACTIONS*** |  |  |  |  |  |
| Location, Urgency*CPT3 Score | Inpatient, non-emergency | 0.446 | 45.9 | - | - |
|  | Inpatient, emergency | 0.011 | 0.01 | - | - |
| Wound Type*PVD | Clean / Contaminated | -0.631 | 15.4 | - | - |
|  | Contaminated - Dirty | -0.886 | 39.5 | - | - |
| Wound Type*SIRS/Sepsis | Clean / Contaminated | -0.194 | 2.63 | - | - |
|  | Contaminated - Dirty | -0.414 | 15.1 | - | - |
| Wound Type*Log(total operation in hours) | Clean / Contaminated | 0.130 | 8.92 | - | - |
|  | Contaminated - Dirty | -0.099 | 4.50 | - | - |
