# Supplementary material for: The Surgical Site Infection Risk Score (SSIRS): A Model to Predict the Risk of Surgical Site Infections
Source: PLoS One. 2013 Jun 27;8(6):e67167. doi: 10.1371/journal.pone.0067167 (PMC3694979; doi:10.1371/journal.pone.0067167)
Supplement: Table S4 — SSI Risk Score (SSIRS). (DOC) [file pone.0067167.s005.doc]

**Table S4:** SSI Risk Score (SSIRS)

| **FACTOR** | **POINTS** |  | **FACTOR** | **POINTS** |
| --- | --- | --- | --- | --- |
| ***WOUND TYPE*** |  |  | ***OR LOCATION and URGENCY*** |  |
| Clean ^a^ | 0 |  | Outpatient^e^ | 0 |
| Clean/Contaminated ^b^ | 6 |  | Inpatient, non-emergency^f^ | 3 |
| Conataminated / Dirty, Infected ^c^ | 12 |  | Inpatient, emergency^g^ | 10 |
|  |  |  |  |  |
| ***BODY MASS INDEX^d^*** |  |  | ***ASA CLASS*** |  |
| <=20 | -1 |  | ASA-1 | 0 |
| 20.1-25 | 0 |  | ASA-2 | 4 |
| 25.1-30 | 1 |  | ASA-3+ | 7 |
| 30.1-35 | 2 |  |  |  |
| 35.1-45 | 4 |  | ***>1 PROCEDURE PERFORMED^h^*** | 2 |
| >=45 | 6 |  | ***GENERAL ANAESTHESTIC^i^*** | 3 |
|  |  |  | ***PATIENT IS A SMOKER^j^*** | 2 |
|  |  |  | ***METASTATIC CANCER^k^*** | 3 |
|  |  |  | ***ON STEROIDS^l^*** | 3 |

|  | **POINTS BY WOUND TYPE** | | |
| --- | --- | --- | --- |
|  | **Clean** | **Clean /**  **Contaminated** | **Contaminated / Dirty** |
| ***OPERATION DURATION (HOURS)^m^*** |  |  |  |
| ≤1/2 | -7 | -8 | -6 |
| 0.51-0.75 | -2 | -3 | -2 |
| 0.751-1.25 | -1 | -1 | -1 |
| 1.251-1.5 | 0 | 0 | 0 |
| 1.51-2 | 1 | 1 | 1 |
| 2.01-3.5 | 2 | 2 | 2 |
| >3.5 | 4 | 5 | 3 |
| ***PERIPHERAL VASCULAR DISEASE^n^*** | 8 | 1 | -2 |
| ***SEPTIC^o^*** | 6 | 4 | 1 |
|  | | | |
|  | **POINTS BY OR LOCATION and URGENCY** | | |
|  | **Outpatient** | **Inpatient, Non-emergency** | **Inpatient, Emergency** |
| ***CPT3 SCORE*** |  |  |  |
| <0.296 | -9 | -13 | -9 |
| 0.296-<0.427 | -7 | -10 | -7 |
| 0.427-<0.586 | -5 | -8 | -5 |
| 0.586-<0.619 | -4 | -6 | -4 |
| 0.619-<0.900 | -3 | -4 | -3 |
| 0.900-<1.100 | 0 | 0 | 0 |
| 1.100-<1.262 | 2 | 3 | 2 |
| 1.262-<1.531 | 4 | 6 | 4 |
| 1.531-<1.593 | 6 | 9 | 6 |
| >=1.593 | 8 | 12 | 8 |

Go to <http://www.ohri.ca/SSI_risk_index> for a webpage to calculate this score and risk. The expected risk associated with each score total can be determined from Figure S1.

**^a^**No inflammation without entry into respiratory, alimentary, genital, or uninfected urinary tract. **^b^**The respiratory, alimentary, genital or urinary tracts are entered under controlled conditions without unusual contamination. **^c^**Open, fresh, accidental wounds OR operations with major breaks in sterile technique or gross spillage from the gastrointestinal tract OR incisions in which acute, nonpurulent inflammation is encountered including necrotic tissue without evidence of purulent drainage. **^d^**Calculated as 700*(weight in pounds)/(height in inches)^2^ **^e^**Patient brought in for scheduled / elective surgery from their home or normal living situation; OR can be done in a single day without the need for overnight stay in hospital. **^f^**Patient brought in for scheduled / elective surgery from their home or normal living situation; OR requires at least one overnight stay in hospital; not-emergency. **^g^**Needs at least one overnight stay in hospital AND both surgeon and anaesthetist state that OR has to be done as soon as possible and no later than 12 hours after admission to hospital. ^h^ An additional operative procedure performed by the same surgical team (i.e. same specialty/service) under the same anesthetic which has a CPT code different from that of the Principal Operative Procedure. **^i^**General anaesthesia was used (instead of epidural, spinal, regional, local, monitored anaesthesia care). **^j^**Patient has smoked cigarettes in the year prior to admission for surgery. Cigars, pipes, chewing tobacco are not included. **^k^**Patients with cancer that: (1) Has spread to one+ sites in addition to the primary site AND (2) with the presence of multiple metastases indicating the cancer is widespread, fulminant, or near terminal. Includes: Acute Lymphocytic Leukemia (ALL): Acute Myelogenous Leukemia (AML); and Stage IV Lymphoma. Excludes: Chronic Lymphocytic Leukemia (CLL); Chronic Myelogenous Leukemia (CML); Stages I through III Lymphomas; and Multiple Myeloma. **^l^**Patient given regular oral or parenteral steroid medication for more than 10 days in the month prior to OR; topical / inhaled / rectal steroids not included. ^m^Hours from first incision to closure. **^n^**Any type of angioplasty (including stent placement) or revascularization procedure for atherosclerotic peripheral vascular disease OR any amputation procedure for PVD; amputations for trauma or a resection of abdominal aortic aneurysms are not counted. **^o^**Patient had SIRS (2+ of: T>38c or <36c; HR>90; RR>22 or PCO2<32; WBC>12 or <4; AG), Sepsis (SIRS and +ve blood culture OR pus), or Septic Shock (Sepsis + organ / ciculatory dysfunction) in the 2 days prior to OR.
