# Supplementary material for: The Surgical Site Infection Risk Score (SSIRS): A Model to Predict the Risk of Surgical Site Infections
Source: PLoS One. 2013 Jun 27;8(6):e67167. doi: 10.1371/journal.pone.0067167 (PMC3694979; doi:10.1371/journal.pone.0067167)
Supplement: Appendix S1 — Definitions used for superficial, deep, and organ surgical site infections. (DOC) [file pone.0067167.s006.doc]

**APPENDIX S1:**  Definitions used for superficial, deep, and organ surgical site infections.

- A **superficial SSI** was counted if only skin and subcutaneous tissue of the incision were involved and any of the following were noted: purulent drainage from the superficial incision; organisms isolated from an aseptically obtained culture of fluid or tissue from the superficial incision; pain, tenderness, localized swelling, redness, or heat with superficial incision being deliberately opened by the surgeon; or diagnosis of superficial incisional SSI by the surgeon or attending physician.

- A **deep incisional SSI** was counted if deep soft tissues were involved and any of the following were noted: purulent drainage from the deep incision but not from the organ or organ space; a deep incision spontaneously dehisced or was deliberately opened by a surgeon when the patient had a fever, localized pain, or tenderness; an abscess or other evidence of infection involving the deep incision was found on direct examination, during reoperation, or by histopathologic or radiologic examination; or a diagnosis of deep incision SSI was made by a surgeon or attending physician.

- An **organ-space SSI** was counted if: purulent drainage noted from a drain that was placed through a stab wound into the organ/space; organisms were isolated from an aseptically obtained culture of fluid or tissue in the organ/space; an abscess or other evidence of infection involving the organ/space that was found on direct examination during reoperation, or by histopathologic or radiologic examination; or diagnosis of an organ/space SSI was made by a surgeon or attending physician.
