# Supplementary material for: The Surgical Site Infection Risk Score (SSIRS): A Model to Predict the Risk of Surgical Site Infections
Source: PLoS One. 2013 Jun 27;8(6):e67167. doi: 10.1371/journal.pone.0067167 (PMC3694979; doi:10.1371/journal.pone.0067167)
Supplement: Appendix S2 — Complete description of all covariates offered to model. (DOC) [file pone.0067167.s007.doc]

**APPENDIX S2:** Complete description of all covariates offered to model

|  | **DERIVATION** | **VALIDATION** | **OVERALL** |
| --- | --- | --- | --- |
|  | **n=181 894** | **n=181 146** | **n=363 040** |
| ***PATIENT DEMOGRAPHICS AND BASELINE*** |  |  |  |
| Mean Age (SD) | 56 (16.9) | 55.9 (16.9) | 56 (16.9) |
| Male | 77 919 (42.8) | 77 138 (42.6) | 155 057 (42.7) |
| **Race:** Black | 17 936 (9.9) | 17 831 (9.8) | 35 767 (9.9) |
| White | 139 299 (76.6) | 138 645 (76.5) | 277 944 (76.6) |
| Other | 24 659 (13.6) | 24 670 (13.6) | 49 329 (13.6) |
| Not institutionalized | 173 920 (95.6) | 173 217 (95.6) | 347 137 (95.6) |
| Smoker | 35 792 (19.7) | 35 526 (19.6) | 71 318 (19.6) |
| **Dyspnea:**  None | 163 063 (89.6) | 162 501 (89.7) | 325 564 (89.7) |
| With 1 flight stairs | 15 355 (8.4) | 15 182 (8.4) | 30 537 (8.4) |
| At rest | 2329 (1.3) | 2321 (1.3) | 4650 (1.3) |
| Do not resuscitate in orders* | 1147 (0.6) | 1142 (0.6) | 2289 (0.6) |
| **ADLs:**  Independent | 171 784 (94.4) | 171 137 (94.5) | 342 921 (94.5) |
| Assistance required from another | 7469 (4.1) | 7442 (4.1) | 14911 (4.1) |
| Total assistance | 2641 (1.5) | 2567 (1.4) | 5208 (1.4) |
| Mean Body Mass Index in lbs/inches^2^ (SD) | 30.1 (8.2) | 30.1 (8.1) | 30.1 (8.2) |

**APPENDIX S2 (continued):** Complete description of all covariates offered to model

|  | **DERIVATION** | **VALIDATION** | **OVERALL** |
| --- | --- | --- | --- |
|  | **n=181894** | **n=181146** | **n=363040** |
| ***PAST MEDICAL HISTORY*** |  |  |  |
| Alcoholism | 4725 (2.6) | 4640 (2.6) | 9365 (2.6) |
| >10% body weight lost in last 6 months | 3334 (1.8) | 3338 (1.8) | 6672 (1.8) |
| Delirious within 48 hours of surgery | 1221 (0.7) | 1284 (0.7) | 2505 (0.7) |
| Unconscious | 83 (0.1) | 111 (0.1) | 194 (0.1) |
| Hemi-, para-, or quadriplegia | 2905 (1.6) | 2775 (1.5) | 5680 (1.6) |
| Previous TIA | 5112 (2.8) | 4984 (2.8) | 10096 (2.8) |
| Previous stroke - with persistent deficit | 4004 (2.2) | 3937 (2.2) | 7941 (2.2) |
| - without deficit | 3683 (2.0) | 3590 (2.0) | 7273 (2.0) |
| Tumour of brain or spinal cord | 596 (0.3) | 618 (0.3) | 1214 (0.3) |
| New diagnosis of CHF in last 30 days | 1655 (0.9) | 1585 (0.9) | 3240 (0.9) |
| Myocardial infarction in last 6 months | 1328 (0.7) | 1208 (0.7) | 2536 (0.7) |
| PCI in past | 10380 (5.7) | 10288 (5.7) | 20668 (5.7) |
| Major cardiac surgery in past | 9775 (5.4) | 9691 (5.4) | 19466 (5.4) |
| Any angina symptoms in last month | 1697 (0.9) | 1674 (0.9) | 3371 (0.9) |
| Hypertension requiring medications | 85227 (46.9) | 84646 (46.7) | 169873 (46.8) |
| Revascularization or amputation for PVD | 6850 (3.8) | 6885 (3.8) | 13735 (3.8) |
| Rest pain or gangrene | 3895 (2.1) | 3867 (2.1) | 7762 (2.1) |
| On ventilator within 48 hours of surgery | 1457 (0.8) | 1503 (0.8) | 2960 (0.8) |
| Severe COPD** | 8912 (4.9) | 8907 (4.9) | 17819 (4.9) |
| Pneumonia on CXR/CT with findings and treatment | 958 (0.5) | 1002 (0.6) | 1960 (0.5) |
| Ascites on exam, US, or CT in last month | 1219 (0.7) | 1185 (0.7) | 2404 (0.7) |
| Varices on EGD or CT | 181 (0.1) | 187 (0.1) | 368 (0.1) |
| Diabetes requiring insulin or oral hypoglycemics | 27809 (15.3) | 27304 (15.1) | 55113 (15.2) |
| Creatinine increasing above 3 mg/dL (264 umoL) | 932 (0.5) | 928 (0.5) | 1860 (0.5) |
| On renal replacement therapy | 2978 (1.6) | 3013 (1.7) | 5991 (1.7) |
| Metastatic cancer*** | 3430 (1.9) | 3317 (1.8) | 6747 (1.9) |
| Steroid in last month for at least 10 days | 5072 (2.8) | 4949 (2.7) | 10021 (2.8) |
| Bleeding diathesis, anticoagulant, or antiplatelet**** | 9570 (5.3) | 9415 (5.2) | 18985 (5.2) |
| pRBC transfusion in last 72 hours | 2057 (1.1) | 1990 (1.1) | 4047 (1.1) |
| Any chemotherapy in last month | 1938 (1.1) | 1819 (1.0) | 3757 (1.0) |
| Radiation/brachytherapy in last 3 months | 1252 (0.7) | 1168 (0.6) | 2420 (0.7) |
| SIRS/sepsis in last 2 days | 13679 (7.5) | 13525 (7.5) | 27204 (7.5) |
| Pregnant | 369 (0.2) | 391 (0.2) | 760 (0.2) |
| Major surgical procedure in last month | 4915 (2.7) | 4790 (2.6) | 9705 (2.7) |

**APPENDIX S2 (continued):** Complete description of all covariates offered to model

|  | **DERIVATION** | **VALIDATION** | **OVERALL** |
| --- | --- | --- | --- |
|  | **n=181894** | **n=181146** | **n=363040** |
| ***SURGICAL INFORMATION*** |  |  |  |
| **Surgical Specialty of Primary Surgeon** |  |  |  |
| General | 115796 (63.7) | 114772 (63.4) | 230568 (63.5) |
| Neurosurgery | 4290 (2.4) | 4347 (2.4) | 8637 (2.4) |
| Otolaryngology | 4149 (2.3) | 4150 (2.3) | 8299 (2.3) |
| Cardiac | 1452 (0.8) | 1440 (0.8) | 2892 (0.8) |
| Thoracic | 1702 (0.9) | 1607 (0.9) | 3309 (0.9) |
| Gynecology | 8273 (4.6) | 8355 (4.6) | 16628 (4.6) |
| Urology | 6230 (3.4) | 6211 (3.4) | 12441 (3.4) |
| Orthopedics | 18011 (9.9) | 18281 (10.1) | 36292 (10) |
| Plastics | 3199 (1.8) | 3171 (1.8) | 6370 (1.8) |
| Vascular | 18787 (10.3) | 18805 (10.4) | 37592 (10.4) |
| Other / Unknown | 5 (0) | 7 (0) | 12 (0) |
| **Surgical Status** |  |  |  |
| Outpatent, non-emergency | 64624 (35.5) | 64695 (35.7) | 129319 (35.6) |
| Inpatient, non-emergency | 98101 (53.9) | 97415 (53.8) | 195516 (53.9) |
| Inpatient, emergency***** | 19169 (10.5) | 19036 (10.5) | 38205 (10.5) |
| **Wound Type** |  |  |  |
| Open wound communicating directly with air | 7516 (4.1) | 7472 (4.1) | 14988 (4.1) |
| Clean****** | 94530 (52) | 94354 (52.1) | 188884 (52) |
| Clean/Contaminated (resp / alimentary / genital / vaginal / urinary) | 63052 (34.7) | 62641 (34.6) | 125693 (34.6) |
| Contaminated / Dirty, Infected | 24312 (13.4) | 24151 (13.3) | 48463 (13.4) |
| **ASA Class** |  |  |  |
| 1 - normal healthy person | 17182 (9.5) | 17635 (9.7) | 34817 (9.6) |
| 2 - mild systemic disease | 82605 (45.4) | 82585 (45.6) | 165190 (45.5) |
| 3-5 - at least severe systemic disease | 82107 (45.1) | 80926 (44.7) | 163033 (44.9) |
| General anesthesia | 166318 (91.4) | 165726 (91.5) | 332044 (91.5) |
| No housestaff | 84895 (46.7) | 85152 (47) | 170047 (46.8) |
| Additional procedure by same surgical team | 67059 (36.9) | 66542 (36.7) | 133601 (36.8) |
| Additional procedure by different surgical team | 6800 (3.7) | 6752 (3.7) | 13552 (3.7) |
| Intraoperative arrest, MI, or unplanned intubation | 207 (0.1) | 204 (0.1) | 411 (0.1) |
| Mean total operation time in hours (SD) | 1.8 (1.5) | 1.8 (1.5) | 1.8 (1.5) |
| NNIS score: 0 | 67191 (36.9) | 67247 (37.1) | 134438 (37) |
| 1 | 81749 (44.9) | 81661 (45.1) | 163410 (45) |
| 2 | 29721 (16.3) | 29087 (16.1) | 58808 (16.2) |
| 3 | 3233 (1.8) | 3151 (1.7) | 6384 (1.8) |

* Does not include advanced directives

** Unable to perform activities of daily living; admitted previously for COPD; requiring chronic bronchodilators: FEV1<75% predicted.

*** Solid tumor at >1 site; ALL; AML; or stage 4 lymphoma

**** With anticoagulant and antiplatelet not discontinued within specified time prior to OR

***** Required both surgeon and anaesthetist to state that surgery needed within 12 hrs

****** No inflammation and respiratory/alimentary/genital/urinary tract not entered
