# Supplementary material for: The Surgical Site Infection Risk Score (SSIRS): A Model to Predict the Risk of Surgical Site Infections
Source: PLoS One. 2013 Jun 27;8(6):e67167. doi: 10.1371/journal.pone.0067167 (PMC3694979; doi:10.1371/journal.pone.0067167)
Supplement: Appendix S3 — Initial logistic model to predict risk of 30-day surgical site infection. (DOC) [file pone.0067167.s008.doc]

**APPENDIX S3:** Initial logistic model to predict risk of 30-day surgical site infection

| **Variable** | **Class Level** | **Estimate** | **Wald χ2** | **Adj**  **Odds**  **Ratio** | **95% CI** |
| --- | --- | --- | --- | --- | --- |
| Intercept |  | -5.879 | 2517.1 |  |  |
| ***A - PATIENT DEMOGRAPHICS AND BASELINE*** |  |  |  |  |  |
| 1/Age |  | -4.076 | 4.3 | 0.02 | (0.00, 0.80) |
| Smoker |  | 0.193 | 41.2 | 1.21 | (1.14, 1.29) |
| >10% body weight lost in last 6 months |  | 0.249 | 16 | 1.28 | (1.14, 1.45) |
| Major surgical procedure in last month |  | 0.176 | 8.3 | 1.19 | (1.06, 1.34) |
| ADLs (vs. Independent) | Partial | 0.201 | 15.7 | 1.22 | (1.11, 1.35) |
|  | Complete | 0.041 | 0.2 | 1.04 | (0.87, 1.25) |
| Body Mass Index |  | 0.011 | 58.8 | 1.01 | (1.01, 1.01) |
| ***B – PAST MEDICAL HISTORY*** |  |  |  |  |  |
| Previous stroke, Persistent Deficit |  | -0.168 | 4.3 | 0.85 | (0.72, 0.99) |
| Revascularization, amputation PVD |  | 0.235 | 15.1 | 1.27 | (1.12, 1.42) |
| Rest pain or gangrene |  | 0.268 | 12.6 | 1.31 | (1.13, 1.52) |
| Severe COPD** |  | 0.124 | 6.5 | 1.13 | (1.03, 1.25) |
| On ventilator within 48 hours of surgery |  | -0.264 | 5 | 0.77 | (0.61, 0.97) |
| Metastatic cancer*** |  | 0.259 | 17.1 | 1.3 | (1.15, 1.46) |
| Steroid in last month for at least 10 days |  | 0.305 | 28.2 | 1.36 | (1.21, 1.52) |
| Bleeding diathesis, anticoagulant, or antiplatelet**** |  | 0.153 | 10.4 | 1.16 | (1.06, 1.28) |
| Radiation/brachytherapy in last 3 months |  | 0.244 | 6.8 | 1.28 | (1.06, 1.53) |
| SIRS/sepsis in last 2 days |  | 0.228 | 27.5 | 1.26 | (1.15, 1.37) |
| ***C - SURGICAL INFORMATION*** |  |  |  |  |  |
| Surgical Status (vs Outpatient, non-emergency) | Inpatient, non-emergency | 0.693 | 229.3 | 2 | (1.83, 2.19) |
|  | Inpatient, emergency | 0.935 | 282.7 | 2.55 | (2.28, 2.84) |
| Open wound communicating directly with air |  | 0.213 | 16.4 | 1.24 | (1.12, 1.37) |
| Wound Type (vs Clean) | Clean / Contaminated | 0.727 | 547.1 | 2.07 | (1.95, 2.20) |
|  | Contaminated - Dirty | 1.07 | 743.1 | 2.92 | (2.70, 3.15) |
| ASA Class (vs 1) | 2 (Mild Disturbance) | 0.367 | 26 | 1.44 | (1.25, 1.66) |
|  | 3+ | 0.563 | 57.3 | 1.76 | (1.52, 2.03) |
| General Anaesthesia |  | 0.288 | 15.2 | 1.33 | (1.15, 1.54) |
| No Resident |  | -0.167 | 38.9 | 0.85 | (0.80, 0.89) |
| Additional procedure by same surgical team |  | 0.336 | 162 | 1.4 | (1.33, 1.47) |
| Additional procedure by different surgical team |  | 0.226 | 22 | 1.25 | (1.14, 1.38) |
| Log (total operation in hours) |  | 0.638 | 951.3 | 1.89 | (1.82, 1.97) |
