# Supplementary material for: The Surgical Site Infection Risk Score (SSIRS): A Model to Predict the Risk of Surgical Site Infections
Source: PLoS One. 2013 Jun 27;8(6):e67167. doi: 10.1371/journal.pone.0067167 (PMC3694979; doi:10.1371/journal.pone.0067167)
Supplement: Appendix S4 — The CPT3 Score. (DOC) [file pone.0067167.s009.doc]

**APPENDIX S4:** The CPT3 Score

| **SYSTEM** | **1^ST^ THREE NUMBERS OF CPT CODE** | **DESCRIPTION** | **CPT3 SCORE** |
| --- | --- | --- | --- |
| Integumentary | 101 | I+D abcess, fluid, hematoma | 1.67 |
|  | 110 | Debridement: skin, subcutanoeuos tissue, muscle, bone | 1.04 |
|  | 119 | Tissue expander insertion | 2.98 |
|  | 143 | Shaving of Epidermal / dermal lesion | 3.36 |
|  | 151 | Epidermal graft | 1 |
|  | 152 | Free Full thickness graft | 1.94 |
|  | 155 | Formation of pedicle | 1 |
|  | 156 | Delay / sectioning of flap | 1 |
|  | 157 | Muscle/Myocutaneous/Fasciocutaneous Flap | 1.13 |
|  | 158 | Graft for Facial Nerve paralysis, Excision excessive tissue | 1.82 |
|  | 159 | Excision, pressure ulcer | 0.48 |
|  | 190 | Mastotomy, drainage of abcsess | 1.62 |
|  | 191 | Excision of breast/nipple lesion identified by radiological marker | 1.91 |
|  | 192 | Excision chest wall tumor, or placement of radiotherapy catheter | 0.89 |
|  | 193 | Mastectomy (partial/total), Mammoplasty, or Breast Reconstruction | 1.07 |
|  | 194 | Breast Surgery NOS | 1 |
| Musculoskeletal | 200 | Incision of Soft Tissue Abcsess | 0.71 |
|  | 201 | Exploration of Penetrating Wound | 1.64 |
|  | 206 | Application/Exchange of External Fixation | 1 |
|  | 208 | Replantation of Limb/Digit | 4.06 |
|  | 209 | Tissue Grafts (Bone, Cartilage, Other), unlisted MSK Procedure | 1.25 |
|  | 210 | TMJ Arthrotomy, Excision of Bone/Tumor of Mandible/Facial Bones | 1.24 |
|  | 211 | Repair/Reconstruction of Facial/Mandible Bone Fracture/Dislocation | 1.27 |
|  | 212 | Reconstruction of Mandible/Maxilla with Graft/Implane, Osteotomy/Osteoplasty Facial/Orbital Bones, | 0.50 |
|  | 213 | Open Treatment Zygomatic/Malar/Nasomaxillary/Orbital Blowout Fracture | 1.81 |
|  | 214 | Open Treatment of Orbital (non-blowout)/Palatal/Maxillary/Mandibular/Hyoid Fracture or Craniofacial Separation | 0.27 |
|  | 215 | Incision and Drainage of Neck/Thorax | 0.76 |
|  | 216 | Resection of Rib/Sternum, Hyoid Myotomy | 1.06 |
|  | 217 | Repair of Rib/Sternum Defect (including pectus excavatum/carinatum) | 0.52 |
|  | 218 | Repair of Rib/Sternum Fracture | 1.16 |
|  | 219 | Excision, tumor, soft tissue of back or flank | * |
|  | 220 | Incision and Drainage of Deep Abcess, Posterior Spine | 0.50 |
|  | 221 | Partial Excision, Vertabrae | 1 |
|  | 222 | Osteotomy, Spine | 0.49 |
|  | 223 | Repair of Verterbral/Odontoid Fracture/Dislocation | 1 |
|  | 225 | Percutaneous Vertebroplasty and Vertebral Arthrodesis | 0.33 |
|  | 226 | Single Posterior Vertebral Arthrodesis Below C2 | 0.47 |
|  | 228 | Multiple Vertebral Arthrodesis, Spinal Instrumentation/Fixation, Total Disc Arthroplasty | 0.67 |
|  | 229 | Unlisted Procedure of Abdomen/MSK System | 1 |
|  | 230 | Shoulder joint exploration, I&D | 2.99 |
|  | 231 | Acriomio/sternoclavicular/Glenohumerol Arthrotomy, Excision, or Curretage | 0 |
|  | 232 | Radical Resection Tumor, Clavicle/Scapula/Proximal Humerus | 1 |
|  | 233 | Removal of Foreign Body or Muscle Transfer, Shoulder | 1 |
|  | 234 | Soft Tissue Repair, Osteotomy, and Prophylactic Treatment of Shoulder Area | 0.21 |
|  | 235 | Open Treatment of Shpoulder Area Fracture/Dislocation | 0 |
|  | 236 | Open Treatment of Shpoulder Area Fracture/Dislocation | 0.54 |
|  | 238 | Arthrodesis, Glenohumeral Joint | 0 |
|  | 239 | Other Procedure, Shoulder | 0 |
|  | 240 | Elbow Arthrotomy or Soft Tissue Tumor Excision of Upper Arm/Elbow Area | 1.41 |
|  | 241 | Excision/Sequestrectomy of Bone/Tumor of Distal Humerus/Radial Head, Or Resection of Elbow Joint | 0 |
|  | 242 | Removal of Foreign Body, Upper Arm/Elbow | 0 |
|  | 243 | Soft Tissue Repair/Arthroplasty, Upper Arm/Elbow Area | 1.41 |
|  | 244 | Osteotomy/Repair/Prophylactic Treatment of Humeral Shaft | 0 |
|  | 245 | Open Treatment of Humeral Shaft/Elbow Fracture/Dislocation | 0.74 |
|  | 246 | Open Treatment of Radial/Ulnar Fracture or Elbow Dislocation | 0 |
|  | 248 | Arthrodesis, Elbow Joint | * |
|  | 249 | Amputation at Humerus or Unlisted Humerus/Elbow Procedure | 0 |
|  | 250 | Forearm Fasciotomy/Tendon Incision/Arthrotomy/Tumor Excision | 3.53 |
|  | 251 | Arthrotomy or Soft Tissue Procedure/Tumor Excision of Wrist/Forearm | 1.80 |
|  | 252 | Soft Tissue Repair, Forearm/Wrist | 0 |
|  | 253 | Tenodesis/Osteotomy/Osteoplasty, Forearm/Wrist | 0 |
|  | 254 | Repair of Non/Malunion, Arthroplasty, Prohylactic Treatment of Forearm/Wrist | 0 |
|  | 255 | Open Treatment of Radial/Ulnar Shaft Fracture | 0 |
|  | 256 | Open Treatment of Distal Radial/Ulnar/Wrist Intra/Extra Articular Fractures | 0.27 |
|  | 259 | Amputation or Disarticulation of Forearm/Wrist | 1 |
|  | 261 | Radical Resection of Tumor, Soft Tissue, Hand/Finger | 1 |
|  | 263 | Repair/Excision of Flexor Tendon, Hand | 0.95 |
|  | 264 | Other Flexor/Extensor Procedure, Hand | 1.53 |
|  | 265 | Muscle/Ligament/Joint Procedure, Hand | 0.99 |
|  | 266 | Open Treatment of Carpo/Metacarpal Fracture/Dislocation | 3.06 |
|  | 267 | Open Treatment of Phalngeal/Metacarpophalangeal Fracture/Dislocation | 0 |
|  | 269 | Amputation of Finger/Thumb, I&D of Pelvis/Hip Joint Area | 0.25 |
|  | 270 | Soft Tissue Procedure or Tumor Excision of Pelvis/Hip Joint Area | 0.92 |
|  | 271 | Other Procedure, Pelvis/Hip Joint Area | 0.68 |
|  | 272 | Open/Closed Treatment of Fracture/Dislocation in Pelvis/Hip Joint Area | 1.07 |
|  | 273 | Tumor Excision or Soft Tissue Repair of Thigh/Knee Joint Area | 0.91 |
|  | 274 | Osteotomy/Arthroplasty/Soft Tissue Repair of Distal Femur/Knee Joint | 0.61 |
|  | 275 | Open Treatment of Thigh/Knee Fracture/Dislocation | 1.20 |
|  | 276 | Tumor Excision/Arthrotomy/Soft Tissue Repair of Distal Leg/Ankle Joint | 1.48 |
|  | 277 | Open Treatment of Distal Tibia/Fibula Fracture, Ankel Arthroplasty | 0.71 |
|  | 278 | Open Treatment of Ankle Fracture, Below Knee Amputation, and Lower Leg Fasciotomy | 1.26 |
|  | 280 | Incision & Drainage of Foot | 0 |
|  | 284 | Open Treatment of Talar/Calcaneous Fracture, and Osteochondral Autograft of Talus | 1 |
|  | 288 | Amupation Foot, Midtarsal or Transmetatarsal | 1.05 |
|  | 298 | Joint Arthroscopy | 0.43 |
|  | 299 | Arthroscopy, Ankle/Hip | 1 |
| Respiratory | 313 | Partial/Total Excision of Pharynx/Larynx | 0.82 |
|  | 314 | Arytenoidectomy, arytenoidopexy, eppiglotectomy | 1 |
|  | 315 | Laryngoplasty or Other Procedure of Larynx | 0.85 |
|  | 316 | Insertion of Laryngeal Speech Prosthesis, or Tracheostoma Revision | 1 |
|  | 317 | Tracheoplasty, Bronchoplasty, or Excision of Tracheal Stenosis/Tumor | 0 |
|  | 318 | Tracheostomy Repair/Closure, or Other Tracheal Procedure | 1.34 |
|  | 320 | Thoracostomy for Rib Resection/Empyema, or Thoracotomy, Minor | 0 |
|  | 321 | Thoracotomy, Major | 0.35 |
|  | 322 | Pneumonostomy, Pulmonary Decortication | 0 |
|  | 323 | Parietal Pleurectomy +/- Decortication | 0.96 |
|  | 324 | Pleural Biopsy or Lung Resection | 0.21 |
|  | 325 | Lung Wedge Resection, Bronchoplasty, Resection of Pancoast Tumor, Empyemectomy, or Pleuredesis | 0.49 |
|  | 326 | Thoracoscopy, Surgical | 0.24 |
|  | 328 | Repair of Lung Hernia/Chest Wall Defect | 2.03 |
|  | 329 | Other Procedure, Lung/Pleura | 1.52 |
| Cardiovascular | 330 | Pericardiotomy/Pericardiectomy, Excision of Pericardial Mass | 0.96 |
|  | 331 | Excision of Cardiac Tumor, Transmyocardial laser revascularization | 1 |
|  | 332 | Ablation Procedure, Cardiac | 1 |
|  | 333 | Exploratory Cardiotomy, Repair of Cardiac Wound/Great Vessels | 0 |
|  | 334 | Repair/Replacement of Cardiac Valve | 0.52 |
|  | 335 | Coronary Artery Repair/Bypass | 0.73 |
|  | 336 | Repair of Cardiac Anomalies/Congenital Defects | 1 |
|  | 337 | Repair of Cardiac Anomalies/Congenital Defects | 1 |
|  | 338 | Repair of Great Vessel Stenosis/Stricture/Anomoly +/- Bypass Graft | 0.45 |
|  | 339 | Pulmonary Artery Repair, Other procedure Cardiac Surgery | 0 |
|  | 340 | Arterial Embolectomy/Thrombectomy by Neck/Thoracic Incision | 1 |
|  | 341 | Arterial Embolectomy/Thrombectomy by Arm/Abdominal Incision | 0.54 |
|  | 342 | Arterial Embolectomy/Thrombectomy by Leg Incision | 0.82 |
|  | 344 | Venous Embolectomy/Thrombectomy | 2.91 |
|  | 345 | Venous Graft/Valvular Repair | 0 |
|  | 348 | Endovascular/Open Repair of Infrarenal AAA/Brachial/Iliac/Femoral Artery | 0.53 |
|  | 349 | Endovascular Graft Placement for Repair of Iliac Artery | 0.60 |
|  | 350 | Direct Repair of Aneurysm/Pseudoaneurysm and Graft Insertion | 0.32 |
|  | 351 | Direct Repair of Aneurysm/Pseudoaneurysm and Graft Insertion, or Repair of AVF | 1.09 |
|  | 352 | Repair of Blood Vessel With/Without Graft | 1.78 |
|  | 353 | Thromboendarterectomy | 0.54 |
|  | 354 | Percutaneous Balloon Angioplasty, Renal Artery/Visceral Arter/Aorta | * |
|  | 355 | Bypass Graft with Vein | 1.29 |
|  | 356 | Bypass Graft Without Vein | 1.20 |
|  | 357 | Exploration/Reoperation of Vessels | 0.38 |
|  | 358 | Postoperative Exploration for Hemmorhage/Thrombosis/Infection, Revision/Repair of Arterial Bypass/Graft | 1.04 |
|  | 359 | Excision of Infected Graft | 0.26 |
|  | 364 | Endovenous Ablation | 0.90 |
|  | 368 | DRIL, and Upper Extremity Hemodyalsis Access (STEAL Syndrome) | 0.49 |
|  | 371 | Venous Anastamosis (Open) | 1 |
|  | 372 | Arterial Revascularization Procedure, Endovascular or Open | * |
|  | 375 | Vascular Endoscopy with Ligation of Perforator Veins | 1 |
|  | 376 | Ligation of Major Artery/Vein | 0.50 |
|  | 377 | Vericose Vein procedure, penile revascularization, or other vascular surgery procedure NOS | 0.76 |
| Lymphatic | 381 | Surgical Procedure of Spleen | 1.03 |
|  | 383 | I&D of Lymph Node Abscess, Lymphangiotomy, or Ligation of Thoracic Duct | 3.23 |
|  | 385 | Pelvic/Peritoneal Lymphadenectomy, Excision of Cystic Hygroma, or other Unlisted Lymphatic Surgery | 0.89 |
|  | 387 | Lymohadenectomy, Other | 0.61 |
|  | 389 | Unlisted Procedure Hemic/Lymohatic System | 2.52 |
| Mediastinum | 390 | Mediastinotomy | 1.01 |
|  | 392 | Excision of Mediastinal Cyst/Tumor | 2.17 |
|  | 394 | Mediastinoscopy, Unlisted Mediastinal Procedure | 0.16 |
|  | 395 | Diaphragm Surgery | 0.95 |
| Digestive | 405 | Lip Excision +/- Reconstruction | 1.47 |
|  | 406 | Lip Repair | 1 |
|  | 407 | Plastic Repair of Cleft Lip/Nasal Deformity | 1 |
|  | 408 | Surgical Procedure on Vestibule of Mouth | 0 |
|  | 410 | I&D of Tongue or Floor of Mouth | 1 |
|  | 411 | Excision of Lesion of Tongue or Floor of Mouth | 0.47 |
|  | 415 | Other Procedure of Tongue or Floor of Mouth | 0 |
|  | 418 | Repair/Resection of Dentoalveolar Structures | 1 |
|  | 421 | Excision of Lesion or Resection of Palate/Uvula | 0.40 |
|  | 422 | Palatoplasty, Repair of Nasolabial Fistula, or Unlisted Procedure of Palate/Uvula | 1 |
|  | 423 | Abscess Drainage or Sialolithotomy of Salivary Glands | 1 |
|  | 424 | Excision of Salivary Glands | 0.35 |
|  | 425 | Plastic Repair or Diversion of Salivary Duct | 1 |
|  | 426 | Ligation of Salivary Duct, or Unlisted Procedure of Salivary Glands/Ducts | 1 |
|  | 427 | I&D of Peritonsilar/Retropharyngeal Abscess | 0 |
|  | 428 | Laryngeal/Pharyngeal Repair or Resection | 0.34 |
|  | 429 | Laryngeal/Pharyngeal Repair or Resection | 0.42 |
|  | 430 | Esophagotomy or Cricopharyngeal Myotomy | 0 |
|  | 431 | Excision of Lesion/Diverticulectomy of Esophagus, or Esophagectomy | 0.98 |
|  | 432 | Laparoscopic Surgery of Esophagus | 0.27 |
|  | 433 | Esophagoplasty or Repair of PEH | 0.64 |
|  | 434 | Repair of Esophageal Wound/Injury/Varices | 0 |
|  | 435 | Gastrotomy or Pyloromyotomy | 1.56 |
|  | 436 | Local Excision/Total/Partial Gastrectomy, Vagotomy or Laparoscopic Gastric Bypass Procedure | 0.60 |
|  | 437 | Laparoscopic Removal/Replacement of Gastric Restrive Device or Laparoscopic Sleeve Gastrectomy | 0.58 |
|  | 438 | Open Gastric Restrictive Procedure, or Formation/Revision of Gastric Anastamosis/Gastrostomy | 1.11 |
|  | 439 | Laparoscopic, Endoscopic or Open Gastric Procedure | 1.43 |
|  | 440 | Enterolysis/Enterotomy/Colotomy/Reduction of Volvulus | 1.56 |
|  | 441 | Enterectomy/Colectomy, Laparoscopic Enterolysis/Enterostomy/Colostomy | 1.53 |
|  | 442 | Laparoscopic Enterectomy/Colectomy/Closure Enterostomy/Colostomy | 1.34 |
|  | 443 | Open Formation/Revision of Enterostomy/Colostomy | 1.52 |
|  | 446 | Enterorrhaphy/Colorrhaphy/Inestinal Stricturoplasty/Closure of Enterostomy or Enteric Fistula | 1.82 |
|  | 447 | Pelvic Exclusion of Small Intestine or Intraoperative Colonic Lavage, Other Unlisted Intestinal Procedure | 1.79 |
|  | 448 | Excision/Repair of Meckel's Diverticulum or Mesenteric Lesion | 1.82 |
|  | 449 | Appendectomy or Other Appendiceal Procedure | 0.96 |
|  | 450 | I&D of Rectal Abscess | 0.80 |
|  | 451 | Open Repair/Excision of Rectum (Partial/Total), Destruction of Rectal Tumor | 1.53 |
|  | 453 | Laparoscopic Proctectomy | 1.57 |
|  | 454 | Laparoscopic Proctopexy, or Unlisted Laparoscopic Procedure of Rectum | 0.61 |
|  | 455 | Open Proctoplasty/Proctopexy, Exploration/Repair of Rectal Injury/Rectocele | 0.99 |
|  | 458 | Closure of Rectovesical/Rectourethral Fistula, Open | 0 |
|  | 459 | Unlisted Procedure, Rectum | 1.53 |
|  | 460 | I&D of Ischiorectal Abscess +/- Fistulotomy | 0.62 |
|  | 467 | Plastic Operation/Repair of Defect in Anus/Sphincter Mechanism | 1.00 |
|  | 469 | Curretage/Cautery of Anal Fissure, Hemorrhoidopexy, or other unlisted anal procedure | 0.29 |
|  | 470 | Open Drainage of Hepatic Abscess or Cyst | 1.22 |
|  | 471 | Partial/Total Hepatectomy | 0.98 |
|  | 473 | Other Surgical Procedure of Live | 0.85 |
|  | 474 | Choledotomy/Cholecysotomy/Cholecystomy/Sphincterotomy +/- Removal of Calculus | 0.70 |
|  | 475 | Laparoscopic Procedure of Biliary Tract | 0.33 |
|  | 476 | Open Cholecystectomy +/- Biliary Duct Exploration | 1.07 |
|  | 477 | Excision Bile Duct Tumor/Choledocal Cyst, Cholecysto/Cheldocoenerostomy, Exploration Biliary Atresia | 1.35 |
|  | 478 | Reconstruction of Extrahepatic Biliary Ducts, U-Tube Hepaticoenterostomy | 1.28 |
|  | 479 | Suture of Extrahepatic Biliary Duct, Unlisted Procedure of Biliary Tract | 0 |
|  | 480 | Placement of peripancreatic Drains, Removal of Pancreatic Calculus | 1 |
|  | 481 | Open Total/Partial Pancreatectomy or Excision of Pancreatic Mass | 1.56 |
|  | 485 | Surgical Treatment of Pancreatic Cyst/Pseudocyst/Stricture | 1.41 |
|  | 489 | Unlisted Procedure of Pancreas | 0.91 |
|  | 490 | Exploratory Laparotomy +/- Drainage of Intra/Extraperitoneal Abscess | 1.32 |
|  | 492 | Open Excision or Destruction of Abdominal Tumor/Cyst, or Staging Laparotomy | 1.02 |
|  | 493 | Laparoscopic Procedure of Abdomen, Peritoneum and Omentum | 0.88 |
|  | 494 | Insertion/Revision of Peritoneal Venous Shunt/Catheter | 0.90 |
|  | 495 | Hernia Repair +/- Mesh, Open | 1.59 |
|  | 496 | Omphalocele/Gastroschesis Repair, or Laparoscopic Hernia Repair +/- Mesh | 0.59 |
|  | 499 | Repair of Abdominal Dehischence, Omental Flap Procedure | 1.42 |
| Urinary | 500 | Nephrolithotomy or I&D of Renal/Perirenal Abscess | 0 |
|  | 501 | Repair of Aberrant Renal Vessels, or Pyelotomy | 1 |
|  | 502 | Partial/Total Nephrectomy, Excision/Ablation of Renal Mass/Cyst | 0.41 |
|  | 503 | Instillation/Removal of Nephrostomy Tube | 1 |
|  | 504 | Pyeloplasty | 1 |
|  | 505 | Repair of Renal Fistula, or any Laparoscopic Procedure of the Kidney | 0.22 |
|  | 506 | Ureterolithotomy/Ureterectomy | 1 |
|  | 507 | Ureteroplasty/Ureterolysis/Ureteroneocystostomy/Ureteropyelostomy/Ureterocalycostomy, or Revision of Urostomy | 1.33 |
|  | 508 | Ureteroenterostomy, Creation of Ileal Conduit or other Urinary Diversion | 1.72 |
|  | 509 | Ureterorrhaphy, Closure of Ureteric Fistula, or Laparoscopic Procedure of Ureter | 1 |
|  | 510 | Cystotomy or Drainage of Perivescical Space Abscess | 1.18 |
|  | 515 | Excision of Bladder Tumor, or Partial/Total Cystectomy, or Pelvic Exenteration | 0.75 |
|  | 518 | Cystoplasty, Vesicourethropexy, Cystorrhapy | 0.83 |
|  | 519 | Surgical Repair of Bladder Fistula, or Laparoscopic repair of stress incontinence | 0.28 |
|  | 522 | Cystourethroscopy, with Fulgeration | 0 |
|  | 523 | Cystourethroscopy, with treatment of Ureteric Defect | 0 |
|  | 524 | Cystourethroscopy with urethral valve repair, or Transurethral Incision of Prostate | 0 |
|  | 525 | Transurethral Resection of Bladder Neck | 0 |
|  | 526 | Transurethral Resection or Laser Treatment of Prostate | 0 |
|  | 527 | Transurethral Drainage of Prostatic Abscess | 1 |
|  | 530 | Urethrotomy or Drainage of Periurethral/Perinel Collection | 1 |
|  | 532 | Urethrectomy or Excision/Fulguration of Urethral Lesion | 0 |
|  | 534 | Urethroplasty, Insertion/Removal of Urethral/Bladder Neck Sphincter | 0.18 |
|  | 535 | Urethrolysis, Urethrorrhaphy, Urethrostomy | 1 |
|  | 536 | Dilation of Female Urethra | * |
|  | 538 | Transurethral Destruction of Prostate Tissue or Other, Unlisted Urinary Procedure | 0 |
| Male Genital | 540 | I&D, Penis | 1 |
|  | 541 | Excision Penile Plaque/Foreign Body, Partial/Total Penile Amputation | 1.34 |
|  | 543 | Plastic Operation, Penis | 0 |
|  | 544 | Penile Shunt/Fistulization or Repair of injury | 1 |
|  | 545 | Orchioectomy, Exploration for Undescended Testicles | 0.64 |
|  | 546 | Laparoscopic Orchiectomy/Orchipexy, Insertion Testicular Prosthesis, Repair Torsioned Testes | 3.62 |
|  | 548 | Excision Spermatocele +/- Epidymectomy | 0.97 |
|  | 549 | Epididymovasostomy | 1 |
|  | 550 | Excision/Repair, Hydrocele | 1.83 |
|  | 551 | Scrotal Procedure | 1.48 |
|  | 552 | Vasotomy | 1 |
|  | 555 | Excision/Ligation for Varicocele, Open or Laparoscopic | 0 |
|  | 556 | Vesiculotomy, Vesiculectomy, Excision of Mullerian Duct Cyst | * |
|  | 557 | Prostatotomy | 1 |
|  | 558 | Prostatectomy or other Prostate Surgery using abdominal approach | 0.14 |
| Female Genital | 559 | Intersex Surgery | 1 |
|  | 564 | I&D Female Genital Abscess, Marsupialization of Bartholin's Gland Cyst | 0.40 |
|  | 566 | Total/Partial/Radical Vulvectomy | 1.51 |
|  | 567 | Excision of Bartholin's Grland Cyst | 3.44 |
|  | 568 | Plastic Repair of Introitus/Clitoris/Female Perineum | 1 |
|  | 570 | Colpotomy +/- Destruction of Vaginal Lesions | 3.26 |
|  | 571 | Vaginectomy or Excision of Vaginal Lesion | 1.34 |
|  | 572 | Plastic Repair of Femal Genitourinary Tract | 0.27 |
|  | 573 | Closure of Vaginal Fistula, Vaginoplasty for Intersex State | 1.46 |
|  | 574 | Laparoscopic Colpopexy or Paravaginal Defect Repair | 0 |
|  | 575 | Conization of Cervix, Cervicectomy, Excision of Cervical Stump | 0 |
|  | 577 | Trachelorrhaphy | 1 |
|  | 581 | Myomectomy, Excision of Fibroid Tumors, TAH, | 0.81 |
|  | 582 | Radical TAH, or Vaginal Hysterectomy | 0.50 |
|  | 583 | Endometrial Cryoablation | 1 |
|  | 584 | Uterine Suspension | 1 |
|  | 585 | Laparoscopic Hysterectomy or other, unlisted uterine procedure | 0.52 |
|  | 587 | Salpingectomy, Salpingo-oophrectomy, Salpingostomy, or Tubo-tubal anastamosis | 0.84 |
|  | 588 | I&D Ovarian Cyst/Abscess, or Transposition of Ovaries | 1 |
|  | 589 | Radical BSO, Resection/Debulking of Ovarian Mass, oophrectomy, Staging Laparotomy | 0.81 |
| Maternity Care | 591 | Surgical Treatment of Ectopic Pregnancy, Open/Laparoscopic | 0.54 |
| and Delivery | 593 | Hysterorrhaphy of Ruptured Uterus | 1 |
| Endocrine | 602 | Excision of Thyroid or Thyroglassal Duct Cyst | 0.13 |
|  | 605 | Parathyroidectomy +/- Transplantation, Thymectomy, Adrenalectomy | 0.30 |
|  | 606 | Excision of Carotid Body Tumor, or Laparoscopic Procedure of Endocrine System | 0.65 |
| Nervous | 613 | CraniectomyCraniotomy for Evacuation of Hematoma or Exploration of Orbit | 0.68 |
|  | 614 | Craniectomy/Craniotomy Subtemporal/Subocciptal or for Lobotomy | 0 |
|  | 615 | Other Craniectomy/Craniotomy | 0.37 |
|  | 616 | Excision of Intracranial Neoplasm, Transection/Ligation of Carotid Artery, Repair of AVM/Aneurysm | 0.35 |
|  | 617 | Repair of Intracranial Aneurysm | 0.53 |
|  | 618 | Surgery for Intracranial Neurostimulator | 0 |
|  | 620 | Elevation of Depressed Skull Fracture | 1 |
|  | 621 | Craniotomy for Repair of CSF Leak/Emphalocele, Reduction of Craniomegalic Skull | 0 |
|  | 630 | Laminectomy, Discectomy, Vertebral Corpectomy | 0.65 |
|  | 631 | Vertebral Corpectomy, Laminectomy with Cordotomy | 1.78 |
|  | 632 | Laminectomy for Spinal Neoplasm/AVM | 0.42 |
|  | 633 | Vertebral Corpectomy for Intraspinal Lesion | 0 |
|  | 637 | Repair of Meningocele or Dural/CSF Leak +/- Laminectomy, Dural Spinal Graft | 0 |
|  | 647 | Neuroplasty, Nerve Decompression, Internal Neurolysis | 1.54 |
|  | 648 | Surgical Repair of Peripheral/Sympathetic Nerve Structure | 0 |
|  | 649 | Nerve Graft/Pedicle Transfer | 1 |
| Auditory | 695 | Radical Mastoidectomy +/- Petrous Apicectomy | 1 |
|  | 696 | Revision of Mastoidectomy, Tympanoplasty | 1.03 |
|  | 698 | Labrynthotomy | 1 |

The unique combinations for the first 3 numbers of CPT codes for without any surgeries in the study cohort are indicated with an asterisk; categories with no observed SSIs but with an expected number of SSIs (based on the initial model) exceeding 0.5 are indicated with a CPT3 Score of “0”; categories with no observed SSIs but with an expected number of SSIs (based on the initial model) lower than 0.5 are indicated with a CPT3 Score of “1”.
